# A Psycholinguistic Look at the Role of Field Dependence/Independence in Receptive/Productive Vocabulary Knowledge: Does it Draw a Line?

**DOI:** 10.1007/s10936-022-09905-4

**Published:** 2022-08-02

**Authors:** Kamal Heidari

**Affiliations:** Victoria University of Wellington, Wellington, New Zealand

**Keywords:** Field dependence, Field independence, GEFT, Receptive/Productive vocabulary

## Abstract

The thrust of this study was to investigate the impact of learning styles in general and Field dependence/Independence (FD/I) in particular on the receptive/productive lexical performance of language learners. It aimed to check whether FD/I learners perform differently on receptive and productive vocabulary tests. To achieve this, first, 94 Iranian language learners were given the Group Embedded Figure Test (GEFT) to determine their learning style; and second, they were put into two groups and were asked to take a receptive and a productive vocabulary test. Having collected and analyzed the data, the study revealed that first, with regard to the receptive test, although FI learners outperformed the FD ones, this outperformance was not significant statistically. Second, for the productive test, a significant difference was found between FIs and FDs with FI learners having a better performance. Third, FI learners acted significantly better in the productive test compared with receptive test. Finally, FD learners performed almost similarly in both receptive and productive tests. The pertinent implications are also discussed.

## Introduction

The point that vocabulary is the masterpiece of every language has become a cliché. Numerous researchers have worked on this pivotal aspect of language which, in turn, has led to different findings on vocabulary. Despite this plethora of research, there are still territories in the domain of vocabulary largely undiscovered. Issues pertinent to receptive/productive vocabulary knowledge in general, and about the role of individual differences in receptive/productive lexical command are among these largely overlooked issues (Heidari, 2019; Hirsh, 2012; Zhong, 2012). On the other hand, it has been asserted that various factors, including but not limited to, teaching methods, classroom atmosphere, teachers’ behavior, and learners’ individual differences might directly or indirectly affect learning and teaching processes (Mackey & Phillip, 1998; Oxford, 2000; Sakar, 2003). Whether or not individual differences bear any impact on receptive/productive vocabulary performance is also far from clarity. In line with this, waring (1999) stated that future studies should be conducted in which the effect of individual differences on the receptive/productive knowledge of learners are investigated. To address this exigency, the current paper tried to shed light on this issue by focusing on the role of two often-researched learning styles, namely, FD/I, as one type of individual differences, in the receptive/productive knowledge of a group of EFL learners. The findings of the study might significantly contribute to more individualized and, as a result, more efficient learning and teaching process in that teaching methods, textbooks, and activities that are more compatible for learners would be used.

## Literature Review

### Receptive/Productive vocabulary

**Fig. 1:**
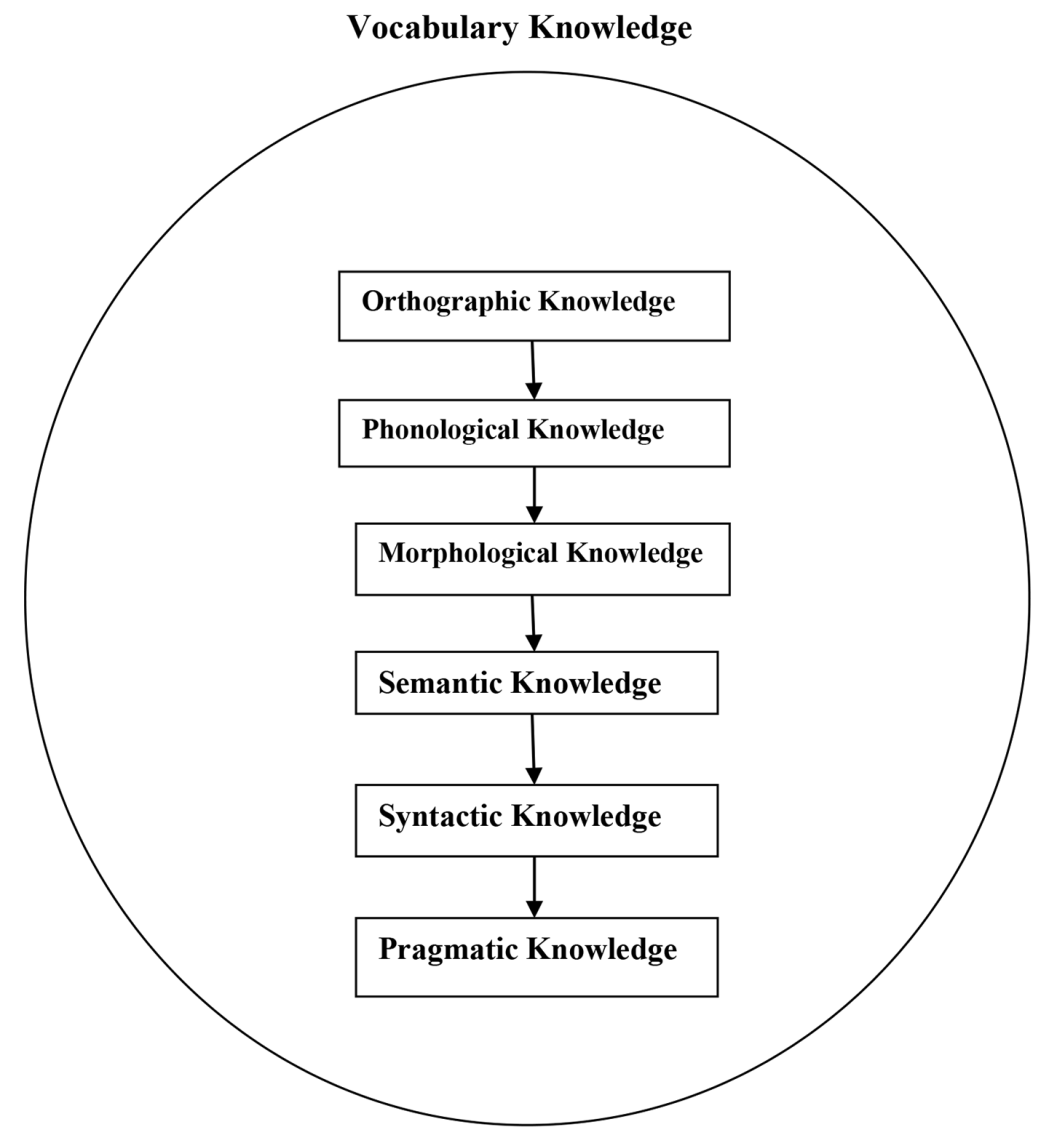
Vocabulary Knowledge Order

Vocabulary knowledge is regarded as a multidimensional construct consisting of a set of sub-constructs (Henriksen, 1999; Henriksen & Haastrup, 1998) including orthographic knowledge (how a word is spelled and written), phonological knowledge (how a word is pronounced), morphological knowledge (the components of a single word), semantic knowledge (meaning of a word, antonyms, synonyms), syntactic knowledge (the position of a word in a sentence, the collocation patterns of the word), and pragmatic knowledge (in which context a specific word should be used). Figure 1 ay reveals this multidimensionality of lexical knowledge and also the common order of their learning more vividly. The point which needs to be pointed out here is that this offered order is not fixed. In other words, different persons depending on different conditions might start with different sub-components of vocabulary knowledge. A learner might, for example, first learn the meaning of a new lexical item without paying any attention to how the word is spelled; while another one first learns the pragmatic aspect of word and then its morphological structure. This proposed order is, however, the most common and logical order for developing vocabulary knowledge which is also directly related to learners’ proficiency level in the sense that the higher the proficiency level of a learner is, the more aspects of lexical knowledge is learned.

The receptive/productive aspect of vocabulary knowledge is also part of the multidimensional nature of vocabulary knowledge. Based on some researchers (such as Abednia & Tajik, 2012), while receptive vocabulary knowledge is part of semantic processing vocabulary knowledge component as it involves learners’ knowledge to process and retrieve the meaning aspect of lexical items; productive knowledge is part of syntactic vocabulary knowledge component in that it entails learners’ knowledge to actively make use of their lexical knowledge in their output. Productive knowledge, however, is related not only to the syntactic knowledge (as Abednia & Tajik (2012) state), but also to the pragmatic knowledge in the sense that it also involves learners’ knowledge about the contextual information of words according to which they should know in what contexts which word is more appropriate to use.

As with the receptive/productive lexical knowledge differences, although there are still unknown angles, some findings have gained consensus. First, Waring (1997) contended that the higher the proficiency level of EFL learners, the higher the likelihood of knowing words receptively and productively. Warring further added that this finding is limited to high-frequency words and in order to be able to generalize it to all words, further studies should be conducted. Webb (2008) also studied the impact of explicit teaching on learners’ receptive and productive knowledge and finally concluded that explicit instruction helps learners know words both receptively and productively. The findings of these two studies, along with some others, imply that receptive and productive knowledge should not be considered to be independent. Instead, they are interrelated and had better be regarded as a continuum. Melka (1997) also argued for the existence of a continuum between receptive and productive knowledge. Melka clarified this continuum contending that it represents increasing degrees of knowledge or familiarity with a word. It means that the more a person knows about a word, the more he/she moves forward from receptive end of the continuum toward the productive end. Notwithstanding the fact that learners’ receptive knowledge is usually larger than their productive knowledge (Melka, 1997; Modrian & Wierma, 2004), learners can, or better to say, should try to extend their receptive knowledge into productive mode and make a balance between the two modes of lexical knowledge (via different ways such as instruction, practice, interaction with others) so that they would be able to use language more efficiently. This point endorses the previously-mentioned point that these two aspects of vocabulary knowledge overlap and are complementary not independent from each other.

To make such equilibrium, some strategies and ways have been offered among which explicit instruction appears to be a good way. Hayashi & Murphy (2009), for instance, stated that based on the findings of studies on receptive/productive knowledge, it seems that learners’ explicit learning of receptive/productive vocabulary knowledge has a positive role in developing their efficient receptive/productive knowledge, which, in turn, leads to their effective language use. Moreover, Webb (2008) also maintained that intentional teaching approach can cause learners to have a balanced development of receptive/productive vocabulary knowledge. Notwithstanding the importance of a balanced development of receptive/productive knowledge, it should also be pointed out that first of all, creating an equal balance between the two types of vocabulary knowledge is out of question mainly due to their different rate of acquisition and development. Nowadays, there is consensus on the point that receptive vocabulary typically grows faster than and before productive vocabulary (Melka, 1997, Ringbom, 1985); and second, the number, amount, and quality of receptive/productive vocabulary knowledge differ across different learners. One reason for such a difference is surely related to individual differences of meaning that different features of learners might directly or indirectly affect their learning both quantitatively and qualitatively. To the best of the author’s knowledge no study has ever dealt with the impact of individual differences, especially FI/D characteristics, on the receptive/productive knowledge of learners. Waring (1999) similarly mention this lack of adequate attention and asks for conducting studies in different contexts To fill such a gap, the present study tried to examine the same issue on a set of Iranian EFL learners.

### Individual differences

Probably the best point to start discussing the issue of individual differences is the fact that there are several factors affecting individuals’ performance. People differ from each other in many terms such as motivation, gender, personality, etc. and each of them can considerably influence their behavior, achievement, and performance. In a similar vein, as far as second or foreign language learning is concerned, learners might be influenced differently by different factors while attempting to master a language (Dornyei, 2009; Griffiths 2008; Naiman et al., 1978; Skehan, 1998). These differences are generally referred to as individual differences. Knowing about these characteristics and how they affect learners’ performance in different strands of language might be advantageous for both students and teachers. In other words, when students know about their individual differences, they would be able to make use of strategies, approaches, and tools that best suit them. Furthermore, teachers’ knowledge about their students’ differences will also help them to provide their learners with the best and most efficient teaching techniques to maximize their teaching effectiveness. Moreover, as Eliason (1995) also rightly put, learners might empower their learning via increasing their awareness of their learning styles and working to develop them further as it can substantially increase their intellectual growth. In keeping with this, teachers can also determine the effective style patterns in their classes and then, devise plans and procedures that are in line with individual learning style preferences.

Individual differences, however, are very wide and are classified into different types. As an example, according to Skehan (1998), language learning process is likely to be affected by all or some of these four main areas: (1) learning styles (2) learning strategies (3) language aptitude and (4) motivation. Each of them, in turn, consists of a set of subcategories. Learning styles, for instance, comprises factors such as FD/I, thinking/feeling, ambiguity tolerance/intolerance, etc. Owning to the fact that considering all of them in a single study is neither practical nor logical, the present study focused on learning styles in general and FD/I in particular.

Learning style is generally defined as the link between personality and cognition (Keefe, 1979). Some researchers (Brown, 1994; Chastin, 1988; Wyss 2002), however, regard this link as the definition of cognitive style arguing that learning style is a sub-type of cognitive style which is restricted to pedagogical contexts. Whether to consider them separate or the same does not seem to make any salient difference in that both terms have the interplay of personality and cognition as their basic characteristic and their difference lies merely in the context domain. In this paper, for the sake of clarity, the term learning style is used. Witkin (1973) simply describes learning styles as the way individuals try to approach, process, and solve a situation, problem, or phenomenon. According to this definition, learning styles deal with the process and not product of activities. One point about this Witkin’s statement, however, is that learning styles not only affect the process directly, but also influence the final product of individuals’ performance. It means that regardless of the ultimate achievement, learning styles of determine how a specific person approaches a problem, issue, or situation and as a result, this differences in dealing with a situation might lead to differences in the final achievement.

**Fig. 2:**
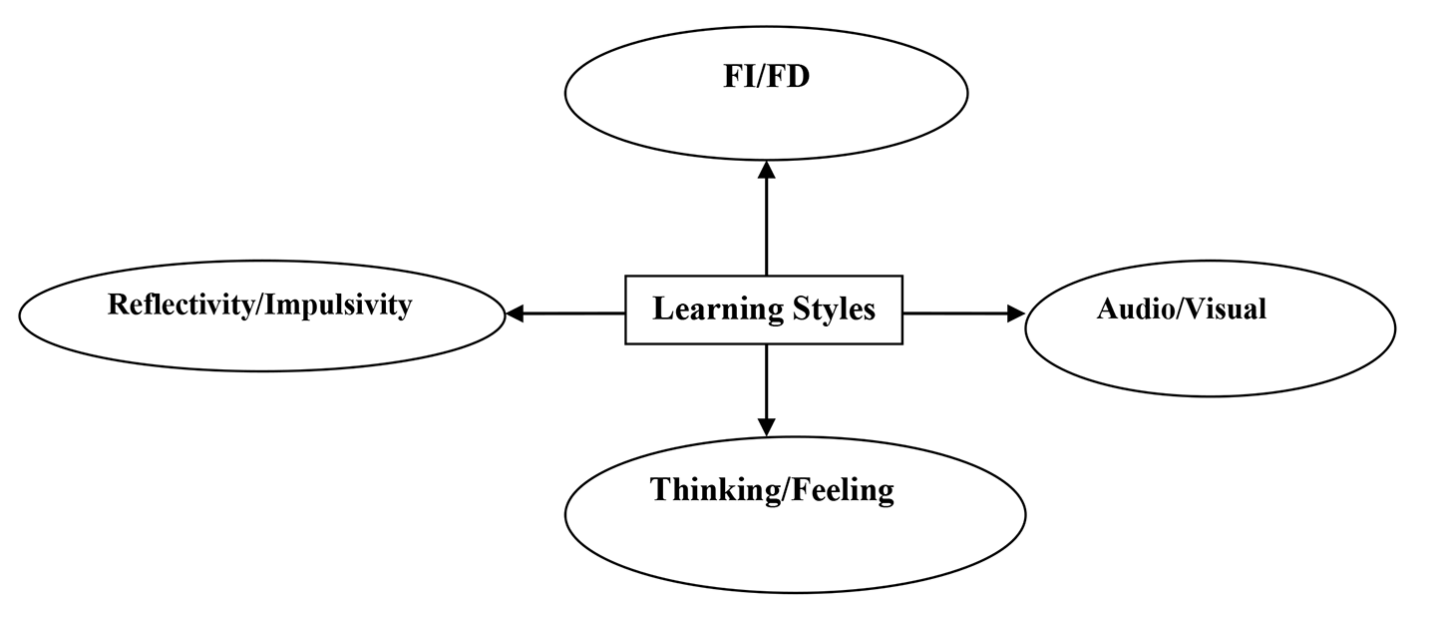
Learning Style as an Umbrella Term

The important point as to learning style is that it should be considered as an umbrella term consisting of a set of components. Wyss (2002), similarly, postulated that there are as many learning styles as there are individuals and the practical implications of learning styles are numerous especially for teaching-learning interactions (P. 1). Figure 2 can more vividly represent the general concept of learning style.

The terms FI and FD have generally been defined as two types of learning styles that illuminate the way people perceive and process information (e.g., Brown 1994; Ellis, 1985; Kang, 1999). To be more detailed, FD learners usually look at the whole of a specific task which contains many items. These learners have difficulty in focusing on a particular item when it is embedded within other items. In contrast, FI learners can easily focus on particular items and are not distracted by other items in the background or context (Gollnick & Chein, 1994). Additionally, FD individuals are globally-oriented meaning that they are more sensitive toward the environment. On the contrary, FI learners are more analytically-oriented in the sense that they are mostly indifferent to the surrounding and prefer to stay independent. Zhang (2004) refered to the source of cues used by individuals to define FI/D terms stating that FI persons make use of internal cues for organizing a conduct while FD ones actively use external cues for thinking, processing, and organizing a conduct.

**Fig. 3:**
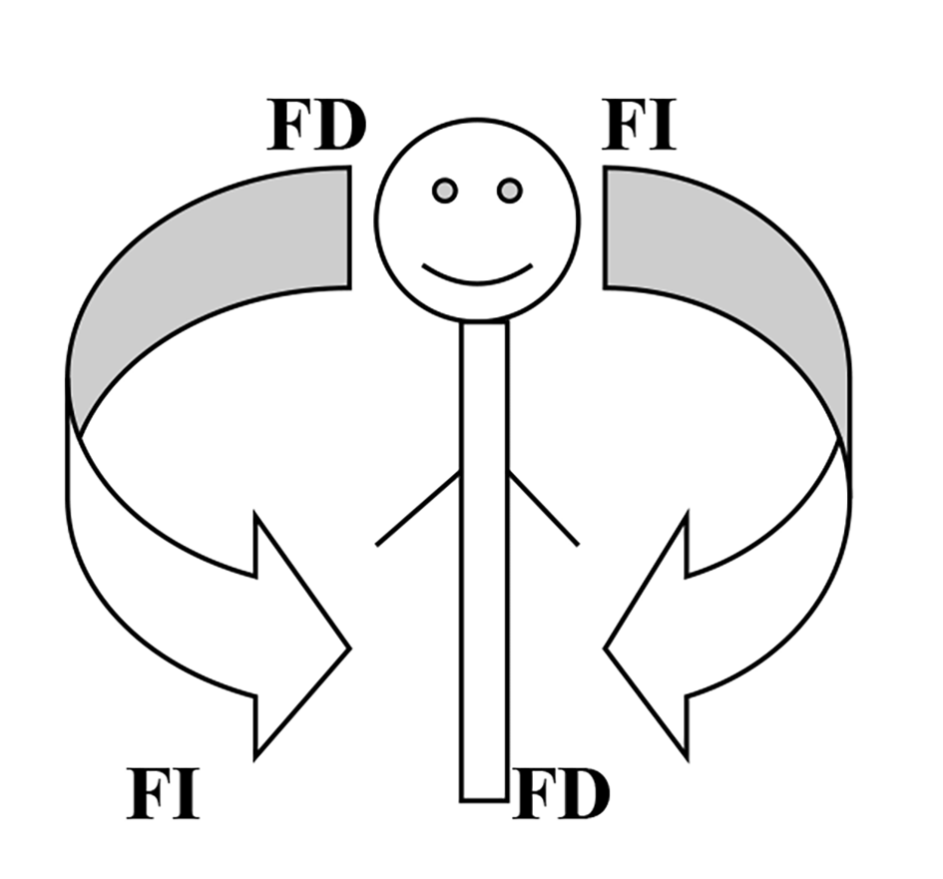
FI/D Relationship

Regarding the impact of these two types of learning style on individuals’ performance in academic contexts, some studies can be found in the literature. For example, it has been concluded in some studies that while FI learners are more likely to outperform FD ones in academic contexts where the focus is on form, analysis, and drills, FD learners usually outperform their FI counterparts in the contexts in which meaning, use, and interaction are emphasized (Ellis, 1994; Skehan, 1998; Yousefi, 2011) took a more or less moderate position maintaining that although both types can be regarded as efficient tools in educational contexts, and although FI learners have been found to be more successful in achieving most of intellectual tasks especially those which require elaborate analysis, process, and attention, it does not necessarily mean that FD learners cannot perform adequately on these tasks. Some points should be mentioned here. First, these findings are all relative and not absolute as not only they have been resulted from a limited number of participants so that they should be approached cautiously, but also, they are resulted in specific contexts with specific contextual factors including specific tasks. Second, terms FI and FD should not be considered absolute either. In other words, FI and FD are better to be taken along a continuum meaning that a single person, depending on a specific context, might be FD, FI, or even field intermediate Fig. 3). Mancy & Reid (2004) have categorized learning styles into three and not two sub-types of field-independence, field-dependence, and field intermediate (also sometimes referred to as field neutral). This taxonomy is highly related to the following figure implying that learners processing ability is flexible and it does not stand to reason saying that one learner is absolutely FI or another learner *Y* is completely FD. Rather, it is better to state that one learner is FI-oriented and another learner is FD-oriented. The term field neutrality or intermediate also implies the same point. It does not mean that a person is neither FI nor FD. Instead it indicates that in some situations a learner might become far from his/her dominant learning style and takes a more moderate style to cope with a specific context.

All taken together, as it was already stated, knowing about learners’ learning styles might be a great help to teachers as they can match their teaching methods with their learners’ learning styles so that teachers’ teaching effectiveness and learners’ learning would maximize. Furthermore, As Brown (1994) also maintained, by creating such a match, learners’ motivation, performance, and achievements would also increase. The point which, however, needs to be noticed is that although learning styles, especially FI/D types, have been extensively investigated as far as language learning is concerned, some gaps are noticeable in the related literature. One is the lack of even a single study (to the best of the author’s knowledge) dealing with the effect of FI/FD on receptive/productive knowledge. Besides, the same problem is even more considerable in EFL contexts where, due to the lack of language use in everyday communications, investigating vocabulary from different angles such as learning styles can effectively influence learners’ performance. To fill such lacuna in the literature, this paper tried to shed light on the impact of FI/D characteristics of a number of Iranian EFL learners on their receptive/productive lexical knowledge.

## Method

### Participants

94 Iranian EFL learners from different language institutes participated in the study. They were both male (43) and female (51) with the age range of 17 to 26 years. According to the language institute placement test (Top Notch Placement test by Saslow & Ascher 2006), the language learners were all upper intermediate learners with almost the same years of English learning (hence, the intervening variable of years of learning could largely be controlled). The learners were required to take three tests: GEFT, a receptive test, and a productive test. Moreover, with regard to ethical issues, a brief summary about the purpose of the study was explained to the participants so that not only their informed consent was obtained, but also negative feelings such as anxiety during the testing sessions as well as cheating or wild guess for a higher score were minimized. Furthermore, in order to hinder any probability of data pollution factors including hawthorn and halo effects, the researcher did not provide them with in-depth explanations on the study.

### Instruments

To gather data, three instruments were used. The first one was the Group Embedded Figure Test (GEFT) that is frequently used to determine the learning styles of individuals. The test, developed by Witkin et al., (1971), asks individuals to identify a geometric figure embedded into a complex figure. It comprises three sections: the first part involves 7 items whose purpose is merely familiarizing individuals with the test. The second and third sections of the test include 18 items (each Sect. 9 items) which are scored dichotomously for the final determination of learners’ learning styles. GEFT scoring range is from 0 to 18, with the mean of 11. Thus, according to Luk (1998), learners scoring below the mean are regarded as FD learners and those above the mean are recognized as FI learners. After giving the test, 49 of the participants turned out to be FI and 45 ones were found to be FD. As to the reliability of this test, Witkin et al., (1971) reported a reliability coefficient of 0.82. Elliotte (1995) also calculated alpha coefficient for the same test which was 0.90. Besides, Witkin & Berry (1975) in their study approved the content, face, and construct validity of the test. Panek et al., (1980) also checked the reliability and validity of the test. They reported that it has acceptable split-half reliability and internal consistency. They further added that construct validity of the test is also satisfactory.

The second instrument was a receptive vocabulary test to measure the knowledge of FI and FD participants. It consisted of 40 items in which the English words were given and the participants were required to recognize their native language (Persian) equivalents from given choices. Finally, with regard to the third instrument, it was a productive test in which 40 native language words (Persian language) were given to the participants and they were asked to recall and write their English equivalents. The two tests involved the same words so that any probable effect of different words could be maximally avoided. Besides, they were picked out from the Academic Word List (Coxhead, 2000). The selected words difficulty level was also in line with the participants’ level of proficiency. Finally, they were all content words comprising 13 nouns, 14 verbs, 10 adjectives, and 3 adverbs.

### Data Collection and Analysis Procedure

The data were collected in three sessions with a one-week interval. In the first session, the participants were required to take the GEFT test. Having taken the test, the author divided them into two groups of FIs and FDs. Then, in the second session, the two groups sat for the receptive vocabulary test. Before taking the test, the researcher provided the learners with brief information regarding the test format, items, and how they were expected to take the test. They were given 45 min for taking the test that, given the number of items, was enough. Finally, in the last session, they were seated for the productive vocabulary test. Like the previous test, they were briefly illuminated about the test and were given 45-minute time limit.

Lastly, Statistical Program for Social Sciences (SPSS) in general and two paired t-tests and two independent t-tests were run to analyze gathered data. To put it more clearly, to measure the receptive and productive lexical knowledge of FI and FD groups, one paired t-test was run for each group. Additionally, for examining the existence of any significant difference between the two groups as far as their receptive and productive knowledge were concerned, two independent t-tests were run.

## Results

To present the obtained results, first, the performance of the two groups on receptive/productive vocabulary tests is presented. Two independent t-tests were run to see whether there was any significant difference between the FI and FD groups in terms of their receptive and productive vocabulary performance. Regarding the receptive lexical test, Table 1 presents the descriptive statistics of this test.

**Table 1:** Descriptive statistics of the receptive test

	Codes	N	Mean	Std. Deviation	Std. Error Mean
**Receptive Test**	**FI Group**	49	12.0	2.2	0.40
	**FD Group**	45	11.07	2.1	0.39

According to the table, the mean of the FI group (M = 12.0) in the receptive test is slightly higher than the mean of the FD group (M = 11.07) but this difference is very marginal. Now to see if the two groups performed significantly different on the same test or not, the independent t-test results are presented in Table 2.

**Table 2:** Independent t-test of receptive test

	T	Df	Sig. (2-tailed)	Mean Difference	Std. Error Difference	95% Confidence Interval of the Difference	
						**Lower**	**Upper**
**FI & FD Receptive Test**	− 0.581	93	0.56	− 0.32	0.56	-1.46	0.80

The table reveals that the two groups did not differ significantly in their performance on the receptive test (t = − 0.58, *p >* 0.05). Therefore, it can finally be stated that although FI learners outperform FD ones in the receptive lexical test, this better performance was not, however, statistically significant.

To investigate the performance of the two groups on productive vocabulary test, another independent t-test was run. Table 3 shows the related descriptive statistics.

**Table 3:** Descriptive statistics of the productive test

		Codes	N	Mean	Std. Deviation		Std. Error Mean
**Productive Test**		**FI Group**	49	27.0	3.8		0.68
		**FD Group**	45	12.3	2.5		0.46

The table indicates that the mean of the FI group (M = 27.0) in the productive test was higher than the mean of the FD group (M = 12.3) which, in turn, indicates that FI learners had a better performance compared to FD learners on the productive vocabulary test. Like the previous case, to see whether this better performance was statistically significant, the independent t-test results are presented in Table 4. As the table clearly shows, the difference between the two groups on the productive test was significant (t= -17.5, *p <* 0.001).

**Table 4:** Independent t-test of productive test

	T	Df	Sig. (2-tailed)	Mean Difference	Std. Error Difference	95% Confidence Interval of the Difference	
						**Lower**	**Upper**
**FI & FD Productive Test**	-17.5	93	0.00	-14.6	0.83	-16.3	-12.9

In the next stage of the study, two paired t-tests were run to investigate the performance of FI and FD groups separately on receptive and productive tests. The purpose was to see if they perform better in any of the two tests or not. As with the FI group, Table 5 represents the obtained results.

**Table 5:** Paired t-test on FI group performance

	Mean		Std. Deviation		T	Df.	Sig.
	**Receptive test**	**Productive test**	**Receptive test**	**Productive test**			
**FI Group**	12.0	27.0	2.2	3.8	-1.84	48	0.000

As it is seen from the table, there was a statistically significant difference between the FI group’s performance in terms of their receptive/productive test (t = -1.84, *p ≤* 0.001). It shows that FI learners had a better performance on the productive test (M = 27.0) than on the receptive test (M = 12.0).

Furthermore, to test the same point for FD group, Table 6 representing the paired t-test results is presented.

**Table 6:** Paired t-test on FD group performance

	Mean		Std. Deviation		T	Df.	Sig.
	**Receptive test**	**Productive test**	**Receptive test**	**Productive test**			
**FD Group**	11.7	12.3	2.1	2.5	-23.2	44	0.003

Based on the table figures it is understood that there was not a significant difference between the FD group’s performance in receptive and productive tests (t = -23.2, *p ≥* 0.001). In other words, FD learners performed almost the same on the given receptive and productive tests with a very slightly (but not significant) better performance on the productive test.

## Discussion

One of the findings of the current study was that notwithstanding the outperformance of FI learners, compared to FD ones, in terms of receptive vocabulary test, this better performance was not statistically significant. In addition, for the productive lexical test, unlike the previous case, FI learners showed a better performance compared to FD learners, but also this outperformance was statistically significant. Before discussing the results, it is necessary to point out that very few, if any, studies have ever addressed such a topic directly. However, some studies can be found that are generally and indirectly pertinent to the study findings. For example, regarding those studies that have reported FIs’ better performance, Genesee and Hamyan (1980), Hansen & Stansfield (1981), and also Naiman et al., (1978) have argued that FI learners are typically found to be more successful learners in the classroom contexts in comparison to FD learners. They further added that FI learners usually perform more successfully in advanced stages of learning than earlier stages. This finding is supported by this study as the participants were advanced language learners and the FI learners acted better than FD ones. Carter (1998) also reported that FI learners are better meaning processors and FD ones are better form processors. In addition to these studies, many other works can be found that have supported the view of the better performance of FI learners compared to FD ones in academic contexts (such as Bean 1990; Davey, 1990). In contrast to the above-cited studies, some other researchers such as Condon (1984) and Nelson (1995) reported that FD learners perform better than FI ones in academic contexts. Moreover, Johnson et al., (2000) maintained that FD learners act more successfully in certain components of academic learning mainly due to the fact that they are more flexible and adaptive than FIs.

With regard to the lack of significant difference in the receptive test, some points need to be taken into consideration. First, this finding should not be considered to be only the effect of learners’ learning style. To put it clearly, some other factors such as learners’ motivation (Gardner & Lambert, 1972), and even their gender (Maghsudy, 2007) might bear influence on the performance of learners. Furthermore, since in the receptive lexical tests, lower pressure and load is put on the learners’ minds to retrieve the first language equivalent of given lexical items, both FD and FI learners could achieve the task almost successfully. In contrast, when it comes to productive lexical tests in which the target language equivalent of given words need to be recalled, further cognitive load would be on learners’ minds and according to the related literature that was fully explained and discussed in the earlier sections of the study, FI learners are potentially more capable of handling such a load and achieving the task more successfully.

Another finding of this study was that while FI learners had a significantly better performance on productive test than receptive test; FD learners’ performance on the two tests was not statistically significant. One reason for the significant outperformance of FI learners compared to FD ones on the productive/receptive tests might be because of the cognitive load that FI/D learners might bear. To put it more clearly, Waring (1997) explained that individuals do not have equal capabilities in learning receptive and productive vocabulary. Waring (1997) further added that receptive/productive words require different cognitive loads to learn in the sense that productive lexical items are usually more cognitively loaded than receptive ones mainly owning to the fact that learners should pay attention to more factors (such as spelling, phonetic features, and also pragmatic features) to learn a word productively. In contrast, learning a word receptively does not pose much cognitive load.

Given these points, a question which might arise is that is this cognitive load related to the learning style of individuals or not. Can we, for example, claim that FI learners are more capable than FD ones in terms of cognitive load tolerance or vice versa? To address this question, first some points need to be mentioned about cognitive load concept. Cognitive load has repeatedly been reported to affect learning (Mayer & Moreno, 2003; Pass et al., 2003; Sweller, 2010). It is divided into three sub-types of intrinsic (defined based on the level of interaction between elements), extrinsic (the cognition imposition by instructional designs), and germane cognitive loads (related to working memory abilities) (Pass et al., 2003). From these three types, intrinsic and germane cognitive loads might be related to the current study findings. In other words, FI learners, as it was mentioned in the beginning sections of the study, are considered to be internal-oriented, analytic, and more accurate than FD learners. From these characteristics it can be inferred that FI learners become more cognitively loaded than FD ones who are known to be external-oriented, holistic, and less accurate. From this point, I wish to infer that FI learners’ memory (whether short or long memory) is more powerful than FD learners which, as a result, helps them perform better in test situations. Furthermore, there is consensus that there is a strong positive relationship between learners’ cognitive load capacity and their information processing ability (Cooper, 1998; Sweller, 1988). This point can also be used to justify FI learners’ better performance in comparison to FD ones. More precisely, since FI learners are typically expected to experience more cognitive load than FI ones, it can be claimed that they are also better information processors than FD learners. Similarly, Skehan (1998) maintained that FI learners are generally considered to have greater selective attention and have more abilities to heed important aspects of language. FI learners are more reflective persons in the sense that they think deeply about different aspects of a situation and also about the strategies used. Skehan (1998) also contended that FI learners are better information processors than FDs (P. 239). Therefore, all these points give credence to the study finding that FIs outperform FDs in terms of productive test.

Related to the previous point, another reason for the FIs’ significant performance compared to FDs might be due to their differences in using learning strategies. FI learners are considered to be more analytic and cognitively-loaded. Therefore, they are expected not only to make use of more strategies but to use them more systematically and effectively. This point may hold true for both receptive and especially productive tests as for the latter type of test, learners require to spend more time, energy, and thinking to elicit the intended target equivalent. The better and more use of learning strategies by FI learners, in comparison to FD ones, has been directly or indirectly supported in the literature (for example, Alipanahi & Mohajeri 2014; Frank, 1984; Jamieson & Chapelle, 1987; Liu & Reed, 1994; Schmitt, 1997; Tinajero & Paramo, 1998). Additionally, it has also been reported that productive lexical items are often more fragile meaning that they are more difficult to learn and remember, and, at the same time, easier to forget (Crow, 1986; Weshe & Paribakht, 1996). Therefore, it may be deduced that learners need to invest more time and energy and also resort to more varying strategies to effectively master them. FI learners, then, are more likely to succeed in this mission in that they are more effective strategy handlers.

## Conclusions

This study investigated the effect of learners’ FD/I on their receptive/productive lexical performance. The study revealed interesting findings. First, as far as receptive vocabulary test was concerned, there was no significant difference between the FI and FD learners. However, FI learners had slightly better performance. Second, regarding the productive test, there was a significant difference between the two groups and FI learners outperformed the FD ones. Third, as to the FI learners and their performance on the receptive and productive test, it was revealed that they significantly acted better on the productive test in comparison with the receptive test. The last but not the least finding was that FD learners could perform relatively equally on both receptive and productive tests with a slightly better performance on the productive test.

The study has some pedagogical and practical implications. The most important implication is that recognizing and knowing the learners’ learning style can help teachers to be able to make use of teaching methods, techniques, strategies, tasks, and activities that best suit learners’ learning style which, in turn, maximizes vocabulary teaching and learning. To clarify the point, if a teacher knows, for instance, that his learner is FI, he can not only let them be involved in tasks that require detailed analysis and thinking, but also help them to both raise awareness about the dominant learning style and to work to enhance the less dominant style so that learning can be achieved more effectively. Related to the previous point is the important point that it is not logical to state that FI style is better or worse than FD style. Rather, both types of learning style are good and may positively affect language learning. Therefore, teachers are required to help learners strengthen all learning styles as much as possible. Another implication which can be ascribed to the study is that when teachers recognize their learners’ learning style and help learners to know about it, it can enhance the sense of self-responsibility and regulation in learners. This, in turn, increases the motivation of learners to work more diligently and with more awareness in their learning process.

Finally, it is highlighted that the necessity to conduct studies on receptive/productive aspects of vocabulary especially in terms of individual differences is strongly felt. Not even a single study has ever directly addressed the present article topic. This lack of studies implies a caution about the study findings according to which the results of this study should be approached cautiously and might not be simply generalized to all contexts and for all individuals from different ages, proficiency levels, and genders. It is, therefore, hoped that this study functions as an incentive for other interested researchers to cast more light on this largely uncharted territory in the vocabulary domain of language.
